# Management of Hydatid Cysts in Pregnancy: A Report of Two Cases and a Review of Literature

**DOI:** 10.7759/cureus.46425

**Published:** 2023-10-03

**Authors:** Mehnaz Gul, Iman Younis, Balamurugan Rathinavelu, Najla Saleh Ben Ghashir, Ravi Kanth Seeli, Rabih Hatem Chahine, Muhieddine Abdul Fatah Seoud

**Affiliations:** 1 Department of Obstetrics and Gynaecology, Sheikh Shakhbout Medical City, Abu Dhabi, ARE; 2 Department of Radiology, Sheikh Shakhbout Medical City, Abu Dhabi, ARE; 3 Department of Pathology, Sheikh Shakhbout Medical City, Abu Dhabi, ARE; 4 Department of Thoracic Surgery, Sheikh Shakhbout Medical City, Abu Dhabi, ARE

**Keywords:** high-risk pregnancy, albendazole antihelminthic, hydatid cyst surgery, pulmonary hydatid cyst, hydatid cyst of liver

## Abstract

Human hydatidosis is a parasitic infection by the larval stages of the Echinococcus (E.) that rarely occurs in pregnancy (1/20, 000-30 000). Canines are the definitive host while humans are the accidental host. They most often affect the liver (60%) and lungs (30%). E. granulosus causes cystic echinococcosis and is the most frequent form. E. multilocularis causes alveolar echinococcosis and is becoming increasingly more common. E. infections often remain asymptomatic for years before the cysts grow large enough to cause symptoms. Hepatic and pulmonary signs and symptoms are the most common clinical manifestations. There is no consensus on their management in pregnancy. We report two pregnancies complicated by hydatid disease of the liver and lung, discuss their problematic management, and review the recent literature.

## Introduction

Hydatid cyst (hydatidosis) is a zoonotic disease that occurs throughout the world, particularly in those areas where people are involved in the cattle-rearing profession. The two types are Echinococcosis (E.) granulosus and multicularis. In humans, hydatid cysts are more commonly caused by the larva of the tapeworm E. granulosus while infection with E. multicularis is less frequent but is more serious. The life cycle of E. granulosus alternates between carnivores and herbivores like dogs and sheep and humans are accidental intermediate hosts where the liberated ova burrow through the intestinal mucosa and are carried by the portal vein to the liver to develop into adult hydatid cysts. The liver is the most common site of the infection and most cysts are found in its right lobe. Some ova pass through a capillary sieve and become lodged in any part of the body, including the lung, peritoneum, kidney, brain, mediastinum, heart, bone, and other parts of the body [1]. In adults, the most common organs to be involved are the liver (60%) and lungs (20-30%). The right lung and the lower lobes are more commonly affected. Moreover, cysts in the lungs are usually solitary and mostly unilateral. Bilateral lung involvement occurs in 20% of cases, and multiple cysts are found in 30% of patients. The diagnosis of pulmonary hydatid cyst is usually made using a plain chest X-ray, contrast-enhanced computerized tomography (CECT) scans of the chest, and immunological tests. Radiologically, an intact cyst typically presents as a round or oval homogenous density with sharp margins. The peripheral blood smear may show leucocytosis, eosinophilia, and raised erythrocyte sedimentation rate (ESR) [2]. The mainstay of management is a combination of medical and surgical interventions. Whenever feasible, surgery is recommended. In developed countries, this condition rarely occurs nowadays; however, hydatidosis is still reported in developing countries. Moreover, hydatid cysts rarely occur in pregnant patients where medical and surgical management is more complicated. The purpose of this manuscript is to report on two rare cases of hydatid cysts of the liver and the lungs in pregnancy and their management and to review the literature.

## Case presentation

Case 1: hydatid cyst of the liver in a pregnant patient

A 22-year-old female, Afghani refugee, G4P2A1, presented at 34 weeks + 5 days of pregnancy to our emergency department, complaining of a few months’ history of chronic epigastric and right upper quadrant abdominal pain associated with vomiting. She gave a history of exposure to farm animals in her home country. Abdominal ultrasound showed multiple lobulated cystic masses in the liver, suggestive of hydatid cysts (the largest measured 7.7x7.5x6.8cm).

**Figure 1:**
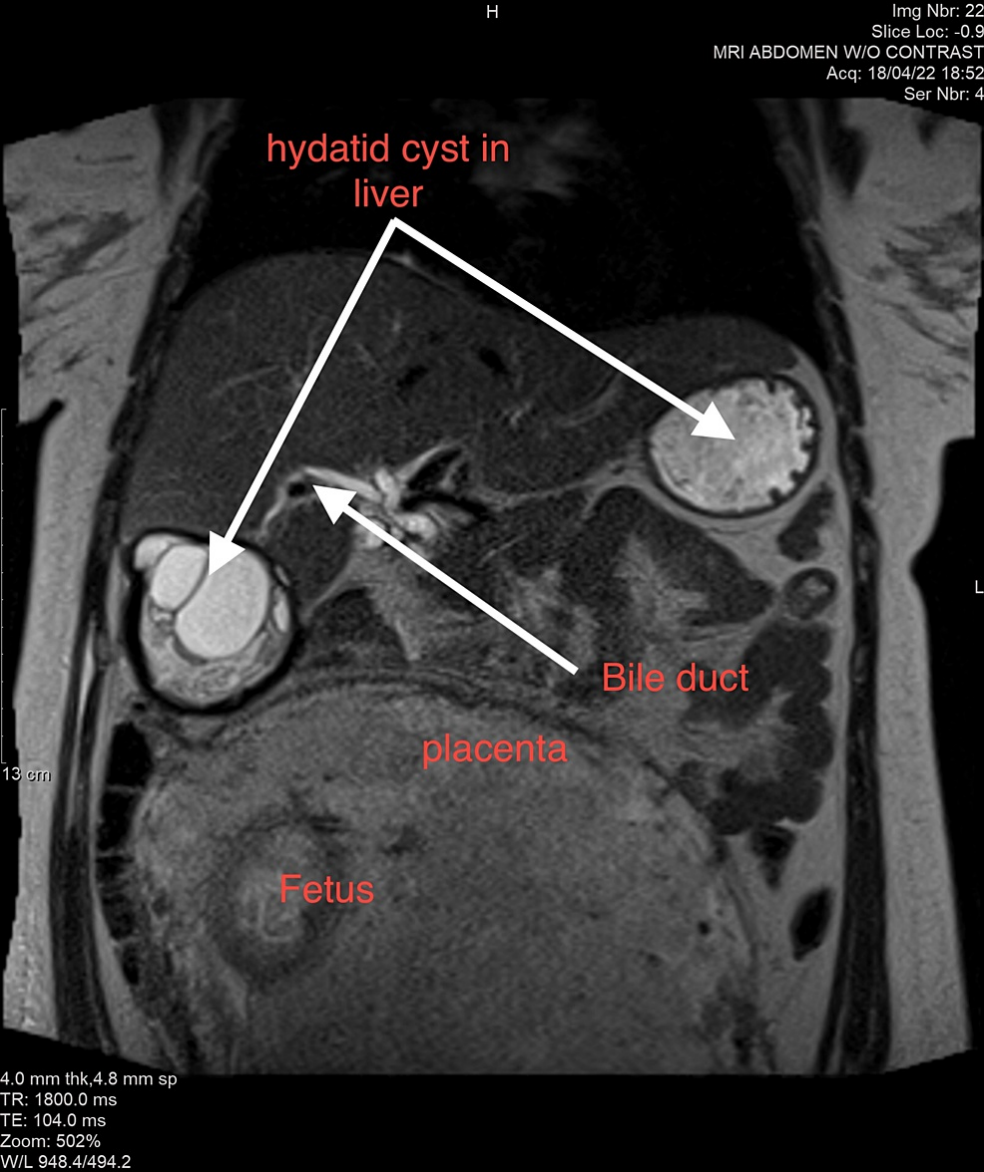
Non-contrast T2 MRI sequence of a pregnant patient, at 34 weeks and 5 days showing two liver hydatid cysts communicating with a biliary tract (double arrows). The fetus and placenta can be seen as well.

Magnetic resonance imaging (MRI) confirmed the presence of a large hydatid cyst in segment 6 of the liver that was very likely communicating with the intra-biliary tree (Figure 1). The serological tests were positive for E. and amoeba antibodies. She had leucocytosis and mildly elevated liver enzymes (aspartate aminotransferase (AST) and alanine transaminase (ALT) 118 and 125 IU/L, respectively). Multidisciplinary team management included a fetomaternal specialist, liver surgeon, radiologist, and infectious disease consultants, who advised delaying antiparasitic treatment till after delivery. She underwent a planned cesarean section at 38 weeks due to breech presentation. Her intrapartum and postpartum course was uneventful. Following the delivery, she continued to receive care at the hepatobiliary clinic in preparation for surgery. However, she left the country due to social reasons before the surgery for the hydatid cyst could be performed.

Case 2: lung hydatid cyst with desaturation

**Figure 2:**
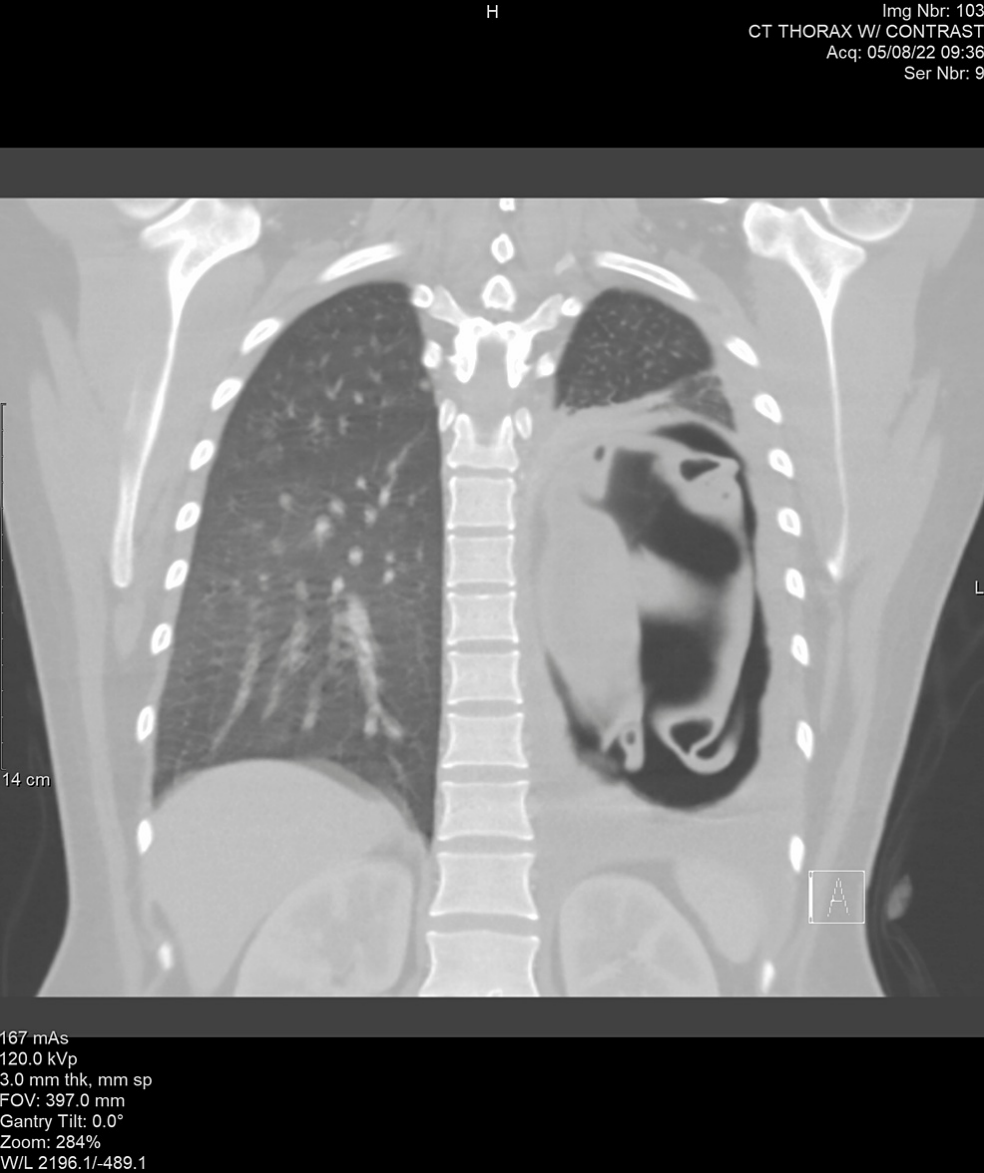
Coronal computerized tomography of a pregnant patient at 21+3 weeks of gestation with a left lung hydatid cyst

**Figure 3:**
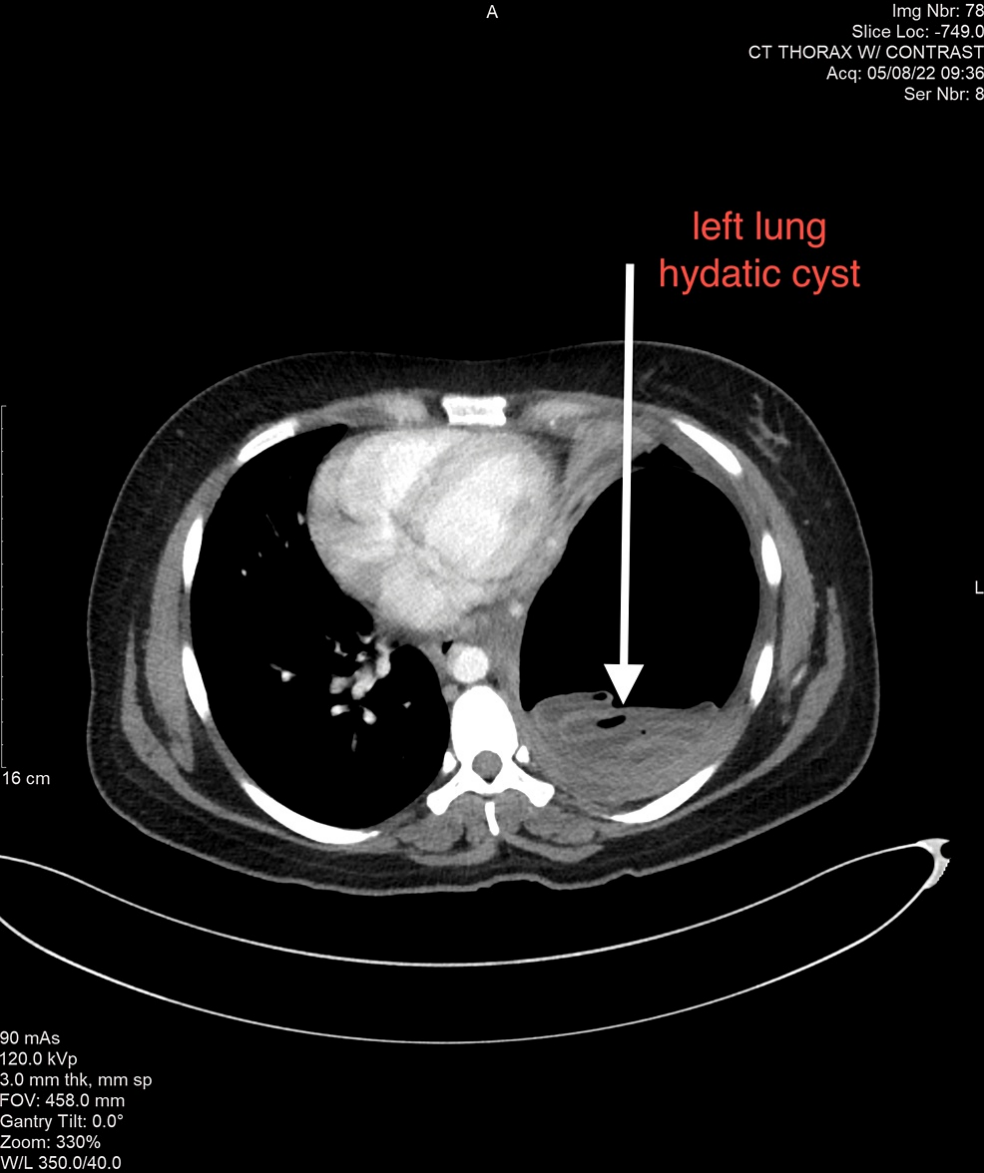
Axial computerized tomography of a pregnant patient at 21+3 weeks with a left lung hydatid cyst

A 26-year-old Pakistani female G2P1A0 at 21+3 weeks of gestation presented to our emergency department with chest pain of three days duration. She had a persistent cough since 2016 that was worsening and was now associated with shortness of breath for the past three months. Additionally, she reported vomiting after coughing and had a low oxygen saturation on arrival (84%). The investigation included a chest X-ray that revealed a 14x13 cm cystic lesion with fluid level in the mid and lower aspect of the left lung, which resulted in a remarkable mediastinal shift to the right side. Serology was positive for E. antibodies. A computerized tomography (CT) scan of the chest showed a 16x10 cm left lower lobe cystic lesion with an internal layering membrane causing a right-sided mediastinal shift consistent with a hydatid cyst (Figures 2-3). In addition, there was a mass effect over the left bronchus with consolidation. Multiple small areas of consolidation were also seen in the right lobe.

**Figure 4:**
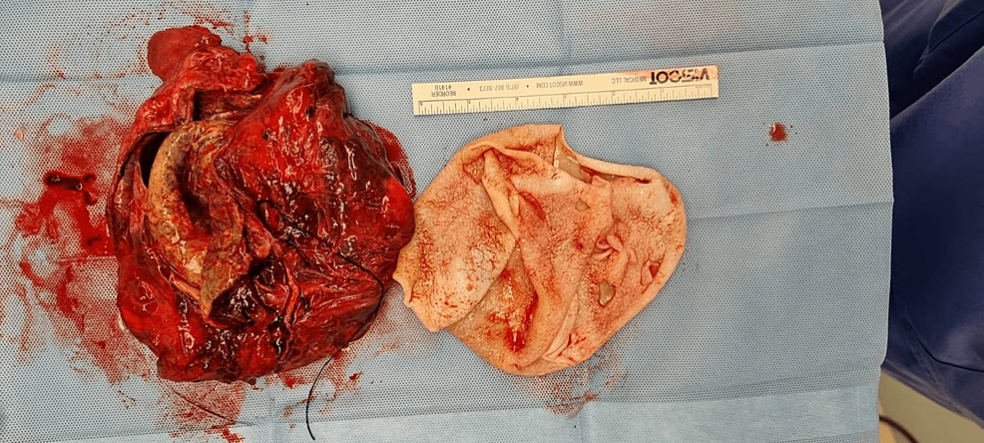
Decorticated pulmonary hydatid cyst with its capsule

Because of the longstanding history of her symptoms, we elected to delay her surgery following a multidisciplinary team meeting, which included the obstetrician, pulmonary surgeon, and infectious disease consultant. She was kept in the intensive care unit (ICU) on oxygen and was started on praziquantel (dose 20 mg/kg twice daily). She had ultrasound-guided drainage of the cyst. Albendazole (400 mg twice daily) was then added to praziquantel. Two weeks following antihelminthic therapy, she underwent thoracic surgery with excision and decortication of the cyst to remove the cyst (Figure 4). Following the surgery, the patient had a flexible bronchoscopy with bronchoalveolar lavage, and she was started on chest physiotherapy.

**Figure 5:**
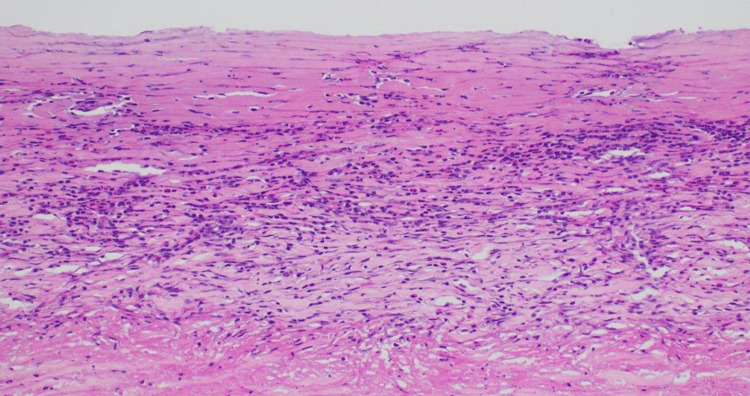
H&E photograph X10 magnification of the lung hydatid cyst showing the outermost fibroinflammatory capsule with eosinophil-rich inflammation H&E: hematoxylin and eosin

**Figure 6:**
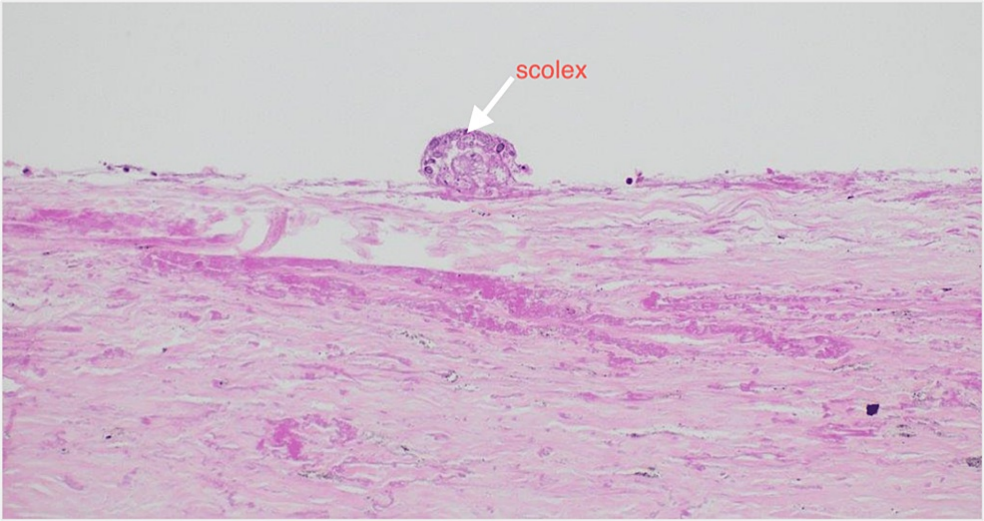
H&E photograph X20 magnification showing a histopathology slide of the lung hydatid cyst with a scolex attached to the cyst wall (arrow) H&E: hematoxylin and eosin

The patient was instructed to continue taking praziquantel 1600 mg bid for two weeks and albendazole 400 mg bid for six months. A follow-up chest X-ray showed that the left upper lobe was fully inflated and had a smaller basal collection. Histopathology was consistent with an E. (hydatid) cyst and the surrounding lung showed chronic organizing inflammation with foreign body granuloma (Figures 5-6). The bronchovascular margin was normal.

The pregnancy progressed without complications until 40 weeks when she had a normal vaginal delivery of a healthy male baby.

## Discussion

Hydatid cysts in pregnancy are, nowadays, exceedingly rare. Management is controversial and consists of a combination of medical treatment followed by surgical excision. However, medical treatment, including albendazole or mebendazole and praziquantel are not safe in pregnancy, especially in the first trimester. Initial treatment by albendazole followed by praziquantel is advised in order to avoid possible weakening and rupture of the hydatid cyst. Preoperative rupture of the hydatid cyst wall may lead to a host of reactions, including fever, urticaria, eosinophilia, potentially anaphylactic shock, and possible dissemination of other cyst contents.

Table 1 summarizes the reported cases in the literature.

**Table 1:** Review of the literature on hydatid disease in pregnancy USG: ultrasonography; ELISA: enzyme-linked immunosorbent assay; RUQ: right upper quadrant pain

Authors	Patient	Clinical Presentation	Diagnosis	Location	Intervention	Recommendations
Srour et al. [3]	27-year-old (5 weeks gestation)	Asymptomatic	Abdominal USG & midline laparotomy	Enlarging splenic hydatid cyst; compressing the liver.	Albendazole, percutaneous drainage, and termination of pregnancy.	Intraoperative diagnosis and CT scan revealed a small hepatic lesion, which dictated the need for albendazole and pregnancy termination.
Goswami et al. [4]	22 years old (term pregnancy)	In labor	USG and ELISA	Multiple abdominopelvic hydatid cysts (largest 7x7 cm^2^).	C-section due to obstructed labor and albendazole.	When a pelvic hydatid cyst causes obstructed labor, a cesarean section is performed with simultaneous removal of the cyst.
Tetik et al. [5]	25-year-old (38 weeks gestation)	Dyspnea with systolic murmur	Echocardiography, MRI, and serologic testing	50- × 60-mm hydatid cyst in the heart (IV septum).	C-section at 39 weeks. Surgical removal 20 days later.	Cardiac involvement is rare (0.5% to 2%). The treatment of hydatid cyst disease is surgical and should not be delayed.
Yilmaz et al. [6]	26-year-old (28 weeks gestation)	Nausea, vomiting, severe headache, left hemiparesis, positive Babinski sign, and right papilledema	Cranial CT, indirect hemaglutination	Right frontotemporal5x6 cm hydatid cyst.	Wide frontoparietal craniotomy with Dowling technique and albendazole. Normal delivery at 39 weeks.	Cerebral hydatid cysts should be treated both surgically and medically. The Dowling technique can be used to remove the cyst without rupturing it.
Ghosh et al. [7]	33 years old (32 weeks gestation)	Severe pruritus, RUQ pain, nausea, vomiting, and low-grade fever	USG, ELISA	7 × 5 cm hydatid cyst in the liver.	Albendazole and PAIR procedure. Normal delivery at 37 weeks.	PAIR followed by long-term oral albendazole for hydatid cysts of the liver diagnosed during pregnancy.
Azlin et al. [8]	26 years old (32 weeks gestation)	Productive cough and SOB for two months	Chest X-ray, CT, ELISA	Lung (left lower lobe) hydatid cyst measuring 9.7 x 7.7 x 12.3 cm.	Albendazole, induction of labor at 35 weeks. Surgical resection 6 months postpartum.	Anthelminthic therapy during 2nd and 3rdtrimester and postnatally in managing pregnant women with pulmonary hydatid cysts.
Our case report. Liver hydatid cyst	22 years G4P2A1, 34+5 weeks gestation	Epigastric and right upper quadrant pain with vomiting.	Ultrasound/MRI/Serology	Large multicystic mass in the right lobe of the liver measuring 7.7x7.5x6.8 cm.	C-section at 38 weeks for breech. Follow up at the hepatobiliary clinic for surgical removal after delivery.	Surgery is the mainstay of treatment for liver hydatid cysts.
Our case report. Lung hydatid cyst.	26 years, G2P1A0 at 21+3 weeks gestation	Cough, shortness of breath, and vomiting	X-ray chest/CT scan/Serology	Left lower lung lobe cystic lesion measuring about 16 x 10 cm.	Praziquantel, IR drainage, albendazole, excision and decortication of cyst.	Surgery when feasible with chemotherapy to avoid recurrence.

One patient with a hydatid cyst of the kidney was managed by the termination of pregnancy in the first trimester [9]. Very few patients with hydatid cysts of the lungs were managed by puncturing the cysts under ultrasound guidance; aspiration of the cystic fluid, injection of hypertonic saline, and re-aspiration of solution without drainage and albendazole therapy (PAIR). However, the treatment of choice for pulmonary hydatidosis remains surgical and may include lobectomy, wedge resection, pericystectomy, and intact endocystectomy. In patients in whom the cysts have already ruptured, thoracotomy and removal of the residual cysts’ wall is recommended. In contrast, the treatment of liver hydatid with drugs alone is appropriate for the management of small cysts (e.g., cysts with a single compartment and diameter <5 cm). Treatment should be administered without interruption. The optimal duration is uncertain; one to three months may be appropriate, however, up to six months may be required. Şahin Ö et al. reported on seven pregnant women with hydatid cysts of the liver [10]. Their presentation included abdominal pain, jaundice, abdominal pain, and an incidental finding. One patient aborted at 16 weeks and the rest were delivered between 30 and 39 weeks, including three premature deliveries at 30, 32, and 34 weeks. Four of the six patients delivered by cesarean section and the rest vaginally. Treatment varied between albendazole alone, surgery and albendazole, and drainage alone. One patient had fever and sepsis while another had an abscess. In one patient, the cyst ruptured spontaneously.

Albendazole is the drug of choice for hydatid disease and is a useful adjunct to surgery. Perioperative drug therapy with albendazole (or mebendazole) reduces the risk of recurrent disease. It also softens the cyst, facilitating removal but may increase the risk of spontaneous rupture [11]. All patients with hydatid cysts treated surgically should receive albendazole (10 mg/kg/day) for six months to prevent a recurrence, which if started in the first trimester means prolonged exposure to the medication. However, the risk of recurrence is as high as 11% if antihelminths are not prescribed post-surgery. Although albendazole has been reported to be embryotoxic and teratogenic in animals, inadvertent exposure of pregnant women to albendazole during mass drug administration for lymphatic filariasis showed no increase in risk for gross congenital anomalies [12].

The postoperative follow-up consists of clinical examination, liver function tests, and chest X-ray once a month for the first three months, which is then continued every three months till the end of the first postoperative year [13]. Pregnancy may limit the type and number of repeated radiologic imaging that can be done especially if it involves repeat CT scanning, which makes the diagnosis and follow-up more challenging.

## Conclusions

Hydatid disease very rarely occurs in pregnancy. The lungs and liver are the two most commonly involved organs. The management of patients remote from delivery involves a combination of medical and surgical treatment. The use of albendazole is the mainstay and concerns about its safety have not been reported in humans.
